# Effects of Surfactants on the Improvement of Sludge Dewaterability Using Cationic Flocculants

**DOI:** 10.1371/journal.pone.0111036

**Published:** 2014-10-27

**Authors:** Yongjun Sun, Huaili Zheng, Jun Zhai, Houkai Teng, Chun Zhao, Chuanliang Zhao, Yong Liao

**Affiliations:** 1 Key laboratory of the Three Gorges Reservoir Region's Eco-Environment, State Ministry of Education, Chongqing University, Chongqing, China; 2 National Centre for International Research of Low-carbon and Green Buildings, Chongqing University, Chongqing, China; 3 CNOOC Tianjin Chemical Research and Design Institute, Tianjin, China; Dowling College, United States of America

## Abstract

The effects of the cationic surfactant (cationic cetyl trimethyl ammonium bromide, CTAB) on the improvement of the sludge dewaterability using the cationic flocculant (cationic polyacrylamide, CPAM) were analyzed. Residual turbidity of supernatant, dry solid (DS) content, extracellular polymeric substances (EPS), specific resistance to filtration (SRF), zeta potential, floc size, and settling rate were investigated, respectively. The result showed that the CTAB positively affected the sludge conditioning and dewatering. Compared to not using surfactant, the DS and the settling rate increased by 8%–21.2% and 9.2%–15.1%, respectively, at 40 mg·L^−1^ CPAM, 10×10^−3^ mg·L^−1^ CTAB, and pH 3. The residual turbidities of the supernatant and SRF were reduced by 14.6%–31.1% and 6.9%–7.8% compared with turbidities and SRF without surfactant. Furthermore, the release of sludge EPS, the increases in size of the sludge flocs, and the sludge settling rate were found to be the main reasons for the CTAB improvement of sludge dewatering performance.

## Introduction

About 3.102×10^9^ tons of municipal wastewater are generated in China in 2007, 49.1% of which need to be treated by some biological processes. Large amount of sewage sludge have been produced in sewage treatment facilities [1]. Raw wastewater sludge contains huge amount of water along with organic solids, which causes problems in transportation, treatment, and disposal [2]. One important stage of sludge treatment prior to disposal is the reduction of sludge volume by sludge dewatering; this process reduces transportation and handling costs [3]. Nevertheless, sludge dewatering remains expensive [4].

Chemicals, such as alum, polymeric ferric sulfate, polyacrylamide, and chitosan, are typically incorporated in sludge to improve the dewaterability [5]–[7]. Flocculants, given at the fixed dosages, are commonly used in conditioning the physical and chemical properties of sludge to improve sludge the dewatering performance, while the polyacrylamide added to sludge is a widely used pretreatment procedure in the wastewater treatment plants (WWTP). The previous reports indicate that the surfactants and polyacrylamide could be used as the dewatering reagents to substantially decrease the moisture content in filter cakes [8]–[9]. Sludge conditioned by the surfactants has gained considerable attention because of their excellent performance in improving sludge dewatering. The surfactants alter the microorganism cell structure by allowing the cell materials to leave the attached surface and simultaneously dissolve this surface in aqueous solutions to improve sludge dewaterability [10]. Huang et al. [11] found that the presence of the surfactants in alum sludge systems was able to improve the sludge quality by reducing SRF and bound water content, as well as increasing settling rate, dewatering rate, and the solid content of the sludge cakes as long as the polyacrylamides were not used. Chu et al. [12] concluded that cationic (CTAB) and anionic surfactants sodium dodecyl sulfate (SDS) enhance the filtration efficiency; however, the former and the latter respectively increase and decrease the consolidation rate, respectively. CTAB and SDS mainly interact with the aggregate surface and interior, respectively. Besra et al. [13]–[14] reported that the mechanism of the reduction of surface tension was responsible for the enhancement of the dewatering kaolin suspensions. The mechanism of the absorption of the complex surfactants in the presence of flocculants exhibits an important function in enhancing the dewatering of kaolin suspensions. In some cases, the reduction in surface tension and the increase in hydrophobicity or a combination of both are able to improve surfactant dewatering [15]. Interfacial tension can be reduced in the sludge system by adding CTAB, a good dewatering performance is obtained [13].

It was also reported that the surfactants can alter the settleability of the activated sludge by changing the release of extracellular polymer substances (EPS). The EPS often comprises polysaccharides, proteins, and DNA, which entrap water and cause high viscosity. EPS is related to the settlement of activated sludge, in which the sludge aggregates are difficult to be packed because of its large size, which would result in interstitially bound water contained in the activated sludge and poor settleability. [16] The evidence mentioned above suggested the need to evaluate the effects of the surfactants on the improvement of the sludge dewaterability using the cationic flocculants. In addition, the effects of the surfactants on improving the actual dewaterability of activated sludge through CPAM are rarely studied. The effects of the surfactant during flocculation on EPS distribution have not been reported.

In this study, the effects of surfactants on improvement of sludge dewaterability by cationic flocculants were investigated. Dry solid (DS) content and the residual turbidity of supernatant were used to evaluate the sludge dewaterability. Specific resistance of filtration (SRF), extracellular polymeric substance (EPS) content of sludge supernatant, floc size, Zeta potential, and settling rate were also measured to explain their changes observed during sludge dewatering. A comparison between sludge dewatering behaviors resulting from flocculation with or without CTAB was made. The causes for the dewaterability improvement after adding CTAB were also elucidated.

## Materials and Methods

### Test materials

Raw municipal sludge samples were collected from the sludge thickener of Dadukou Drainage Co., Ltd. (Chongqing, China). The sludge thickener was just used to store the activated sludge without adding any chemical reagent. The samples were then transported to the laboratory within 30 min after sampling. The samples were subsequently stored at 4°C in the refrigerator; all experiments were completed in 48 h. The initial characteristics of the sludge were as follows: pH 6.80±0.12, Dry solid content (DS) 1.80%±0.36%, total suspended solid (TSS) 55%±9%, mass density (TCOD and SCOD) 0.885 mL·g^−1^. CTAB (molecular formula, C_16_H_33_N(CH_3_)_3_^+^Br^−^; molecular weight, 364.4; ionic type, cationic; aggregation number, 91) was applied to improve dewaterability. CPAM was synthesized in laboratory; the detailed synthesis and characterization of CPAM have been previously reported [5], [17]. The flocculants used in the dewatering tests were CPAM1 (2.0 dL·g^−1^intrinsic viscosity and 30% cationic degree) and CPAM2 (2.0 dL·g^−1^ intrinsic viscosity and 40% cationic degree).

### Flocculation and dewatering experiments

A program-controlled Jar-test apparatus (ZR4-6 Jar Tester, Zhongrun Water Industry Technology Development Co., Ltd., China) was used for sludge dewatering experiments. The dewaterabilities of the sludge flocculated with CPAM alone and with CPAM and surfactant were assessed in terms of residual turbidity of the supernatant, dry solid content, specific resistance to filtration (SRF), settling rate, zeta potential, and floc size. Dewatering tests for waste sludge were carried out at room temperature. Each result was an average of three repeated tests under similar experimental conditions. All the standard deviations were controlled in less than 5%. The pH of the sludge system was adjusted by adding NaOH (1.0 mol·L^−1^) or HCl (1.0 mol·L^−1^). Waste sludge (volume, 500 mL) was transferred into beakers before the required amount of flocculants (CPAM) or the mixture with surfactants (CTAB) was added. The sludge solution was rapidly mixed at 100 rpm for 30 s, followed by slow stirring at 30 rpm for 2 min and sedimentation for 10 min.

### Analytical methods

Proteins and polysaccharides were selected using the method of Raynaud et al. [18] to characterize the composition of EPS. The proteins were determined according to the method of Lowry et al. [19] with BSA as the standard. The polysaccharides were assayed by phenol–sulfuric acid method of Dubois et al. [20] with glucose as the standard. All assays were conducted in threefolds. If the proteins and polysaccharides appeared in the filtrate following the addition of surfactants/flocculants, it was assumed that the EPS was from the activated sludge [21]–[22].

Vacuum filtration was selected to measure the dewaterability of the flocculated sludge. The supernatant of the sludge after flocculation was extracted for the turbidity measurements using a turbidimeter (HACH 2100Q, USA). After rapid agitation, Zeta potential of the supernatant was measured with Zetasizer Nano ZS90 (Malvern, U.K.). Meanwhile, the size of the sludge floc was measured simultaneously with Winner 2000 laser particle size analyzer (Jinan Micro-Nano Technology Co., Ltd., China). The settling rate of the sludge was expressed in terms of the height of the sludge–water interface as a function of time. The sludge sample (volume, 500 mL) was then transferred to a graduated cylinder and allowed to settle after four times of inversion. The height of the liquid slurry interface was noted at a regular time interval without disturbance. The settling rate was the average settling rate calculated in the first 2.5 min of settling time, and the standard deviation was less than 5%.

The flocculated sludge was poured into a Buchner funnel for filtration under a vacuum pressure of 0.06 MPa for 15 min or until the vacuum cannot be maintained in <15 min. The filterability of the sludge was measured by the specific resistance of the sludge. DS was determined by the following equation given as 
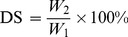
(1)where *W*_1_ is the weight of wet filter cake after filtration, and *W*_2_ is the weight of filter cake after drying at 105°C for 24 h.

*SRF* was calculated as following [21]:
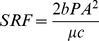
(2)where *P* is the pressure of filtration (N/m^2^), *A* is the filtration area (m^2^), *m* is the viscosity of the filtrate (N s/m^2^), *c* is the weight of solids per unit volume of filtrate (kg/m^3^, *c* = 1/*C*_i_/((100*C*_i_)–*C*_f_)/(100*C*_f_)), *C*_i_ is the initial moisture content (%), *C*_f_ is the final moisture content (%), *b* is the slope determined from the *t*/V_f_(y)–V_f_(x) plot, *V*_f_ is the volume of filtrate (m^3^), and *t* is the filtration time (s). The effect of different dewaterability parameters was shown in Table 1.

**Table 1:** The effect of different dewaterability parameters.

Dewaterability parameters		Effects
Abbreviation	Full name	
DS	Dry solid content	The dry solid content of filter cake after dewatering, indicating the dewatering degree
Turbidity	Residual turbidity of supernate	Representing the quality of flocculation process
EPS	Extracellular polymeric substances	Affecting the distribution of moisture in the sludge particles. Polysaccharide, protein are the main components of EPS
SRF	Specific resistance to filtration	Its value is an important parameter to measure difficulty lever of filtration.
Zeta potential		Charge character of sludge particle surface
Floc size distribution		Representing sizes of sludge flocs, larger floc size indicates the better dewatering performance.
Settling behavior		Representing sedimentation properties of activated sludge

## Results and Discussion

### Effect of surfactant dosage on dewatering performance

**Figure 1:**
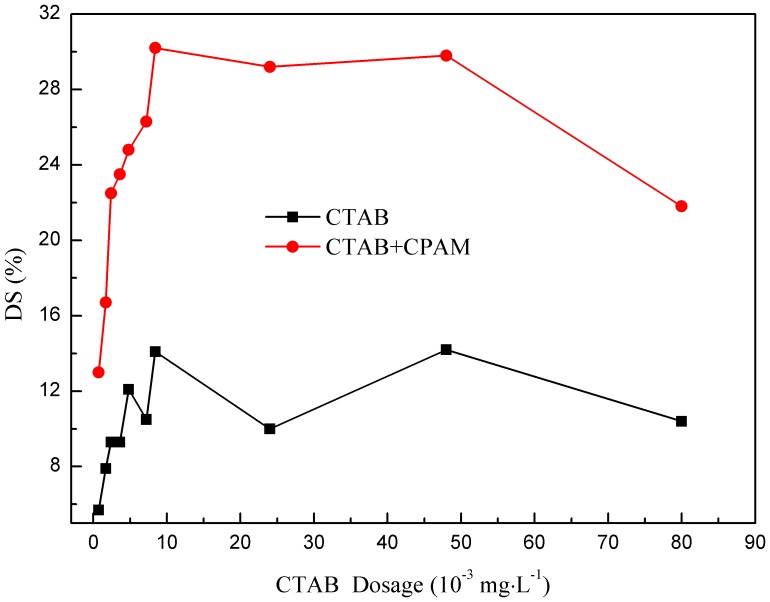
Effect of surfactant dosage on DS.

Figure 1 show the effect of addition of the surfactant on sludge dewatering performance. In addition, no apparent sludge–liquid separation surface is observed, and the supernatant turbidity cannot be measured, as the surfactant merely promoted destabilization and failed to bridge the sludge particles [22].

Mixing the surfactants and flocculants and adding these materials to the sludge sample reduced the volume of dewatered sludge and enhances the DS content. The surfactant significantly improved the sludge dewatering performance in the case of CPAM1 (Figure 1). However, considering keeping the surfactant costs as low as possible at a highest possible DS content, the optimal CPAM1 dosage was 40 mg·L^−1^, while the surfactant dosage was selected at 10×10^−3^ mg·L^−1^ (2 wt% CPAM1), and the CTAB content was kept constant at 2 wt% CPAM1 in the subsequent tests.

### Effect of flocculant dosage on dewatering performance

**Figure 2:**
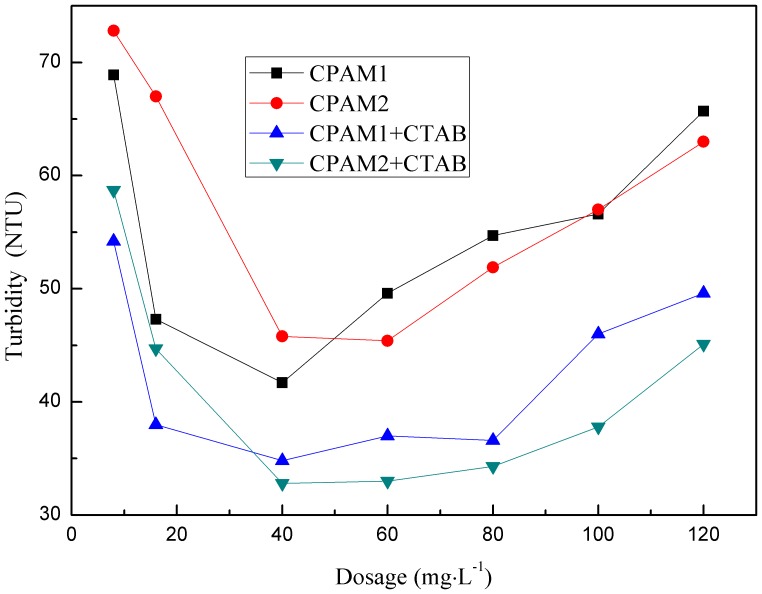
Effect of flocculant dosage on supernatant turbidity.

**Figure 3:**
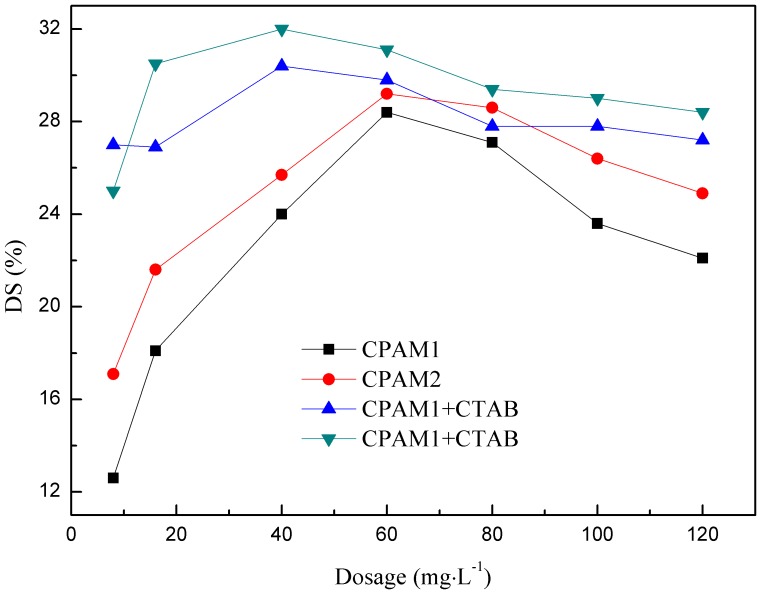
Effect of flocculant dosage on DS content.

Figures 2 and 3 show the effect of flocculant dosage on supernatant turbidity and DS content in the presence and absence of CTAB, respectively. Initially, increasing the flocculant dosage quickly decreases the supernatant turbidity and then sharply increases it. However, DS content was markedly increased and then slightly decreased. The flocculation performance significantly improved upon addition of CTAB during this process; incorporation of CTAB decreased the turbidity of the supernatant and increased the DS content. Compared to not using surfactant, DS increased by 8%–21.2%. An explanation for the obtained results could be as follows: At low dosages, CTAB failed to fully neutralize the negative charge on surfaces of sludge particles; CPAM was unsuccessful in linking or bridging the particles together. The formed sludge flocs are too small, loose, and fragile [23]. Flocculant overdose gradually increased the positive charge of the colloidal system; electrostatic repulsion causes detachment of some segments to other sludge particles. At last, the optimal CPAM dosage was fixed at 40 mg•L^−1^, and the CTAB dosage was 2wt% CPAM. This result was similar to the previous study about enhancing the papermaking sludge dewatering by surfactant [24]. Combined with the role of CTAB and CPAM, the sludge system flocculated by CTAB and CPAM into the slurry system, a good dewatering result could be obtained and the sludge dewatering properties and settlement could be significantly improved.

**Figure 4:**
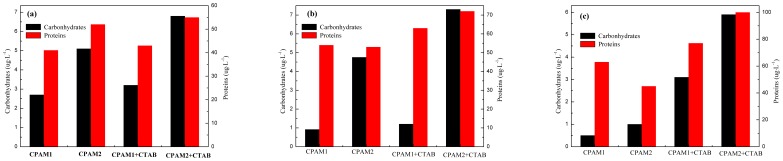
Effect of flocculant dosage (a) 20, (b) 40, and (c) 80 mg·L^−1^ on EPS.

Figure 4 showed that the carbohydrate and protein concentrations in the supernatant gradually increased in the presence of CTAB, but the increase degree of the concentration of carbohydrates and proteins was small. The concentrations of the carbohydrates and proteins in the supernatant increased to 18.6%∼520% and 7%∼122% after addition of CTAB, respectively; a high amount of EPS was dissolved when the sludge was flocculated with a high cationic degree. In the presence of CTAB, the carbohydrate and protein concentrations in the supernatant by CPAM2 at 80 mg/L are 5.9 and 100 µg•L^−1^, respectively.

An explanation could be that the suffusion resulted in the absorption of the external surface of sludge flocs by water molecules, leading to an increase of the amount cell adhesion water, and the formation of thick water film on the protein layer surface [25]. Therefore, the presence of EPS in the sludge flocs increased the difficulty of the sludge dewatering. One possible explanation for the enhanced surfactant-mediated sludge dewatering is the solubilization of EPS in the supernatant; EPS leaves the activated sludge surface and dissolves into water. Reduction of EPS results in a more compact activated sludge under the same mechanical force or at the same dewatering time [26].

### Effect of pH on dewatering performance

**Figure 5:**
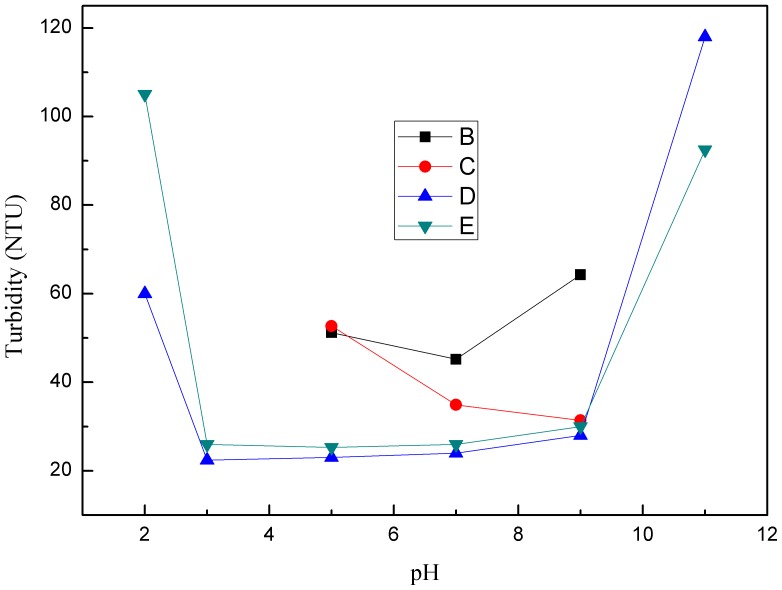
Effect of pH on supernatant turbidity.

**Figure 6:**
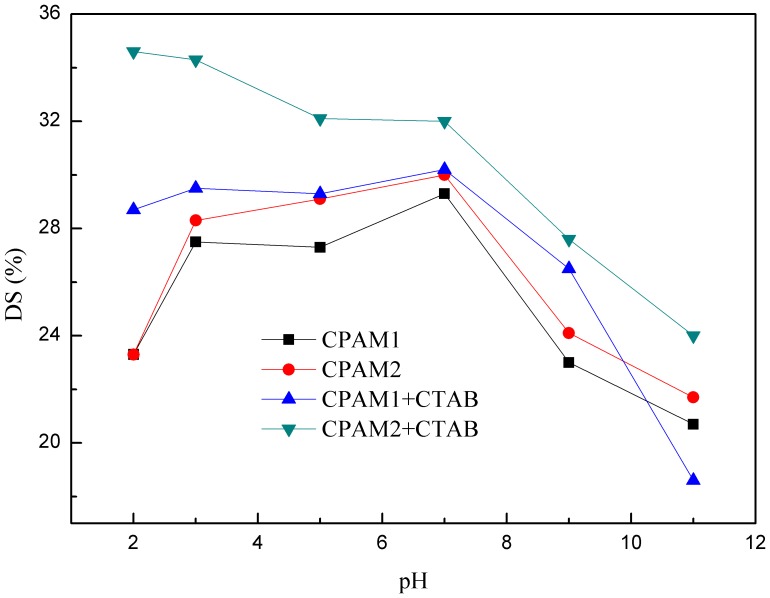
Effect of pH on DS content.

**Figure 7:**
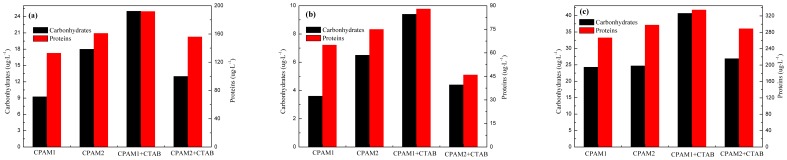
Effects of pH value on EPS at (a) pH = 3, (b) 7, and (c) 11.

Figures. 5–7 illustrate that the changes in pH significantly affect the flocculation performance and sludge dewatering ability. Increasing the pH initially decreased the turbidity of the supernatant and then markedly increased it. Meanwhile, when the pH value was in the range of 2 to 5 and 9 to 11, the residual turbidity of supernate flocculated by CPAM1 and CPAM2 was too high to be determined. However, the same change in pH slightly increased the DS content and decreased thereafter. The optimal DS contents were obtained at pH 6–8 for CPAM+CTAB. Figure 6 shows that the DS content is higher in the presence of CTAB compared with that in the absence of CTAB under an acidic conditions. Strong acid and alkaline conditions deteriorated the formation of the flocs and the performance of the CPAM-treated sludge dewatering. Small flocs were formed and there was little supernatant volume obtained; a blurry sludge–water separation was observed. H^+^ enhanced the positive charges of the sludge surface at low pH, resulting in an increased electrostatic repulsion and colloidal stability [16]. Increasing the specific resistance of sludge to filtration would yield a low DS content. The negative charges on the surfaces of the sludge particles increased at higher pH; these charges could not be fully neutralized by CPAM at a fixed dosage, thereby increasing the repulsive force between the sludge particles and their specific resistances to filtration. Addition of CTAB decreased some negative charges on the sludge surface and reduced the repulsion between sludge particles. The optimum pH for sludge dewatering ranged between 5 and 8 (Figure 6).

Figure 7 shows the changes in EPS concentration in the supernatant after surfactant addition under different pH. High EPS concentrations were released from the sludge particles under acidic and the alkaline conditions, especially for CPAM1, which increased the viscosity of sludge and deteriorated the dewaterability [27]. For example, the concentration of the carbohydrates flocculated by CPAM2 at pH 3, 7, and 11 were 18.0 ug•L^−1^, 6.5 ug•L^−1^, and 24.7 ug•L^−1^, respectively. While the concentration of the proteins flocculated by CPAM2 at pH 3, 7, and 11 were 161 ug•L^−1^, 75 ug•L^−1^, and 298 ug•L^−1^, respectively. The EPS in increasing the concentrations followed a decreasing order: basic>acidic>neutral, because the surfactant was connected between water molecules and the EPS on surfaces of sludge flocs. EPS was released from these surfaces upon stirring. The surfactants also increased the solubility of EPS in water phase, which resulted in an easy dissolution and release of EPS from the surfaces of sludge flocs. The permeability of microorganisms in the sludge was changed by adjusting acid or alkali; the intracellular proteins were denatured and the adhesion force between microorganisms in the sludge particles and the EPS was reduced. The release of the EPS reduced a large number of hydrophilic groups on the surfaces of sludge flocs; the content of the bound water in the sludge likewise decreased, which enhanced the performance of the sludge dewatering. The results indicated that the enhanced dewatering performance was attributed to the reduction of EPS in the sludge upon addition of the surfactants.

The improvement of the dewatering upon basic/acidic and surfactant treatments was attributed to the activated sludge surface left by a portion of EPS; the sludge aggregates are thus easily packed and the water content of dewatered sludge is reduced [16].

### Effect of flocculant dosage on SRF

**Figure 8:**
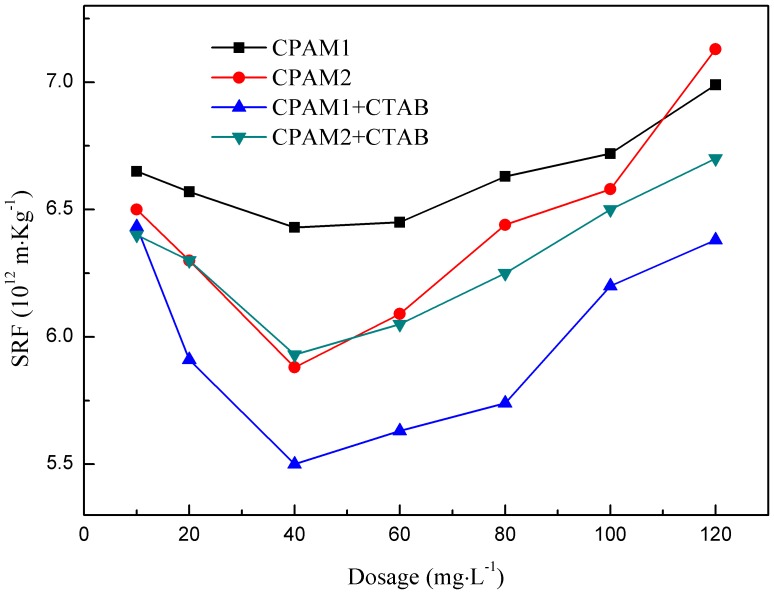
Effect of flocculant dosage on SRF.

Figure 8 shows that the addition of CTAB initially reduced the specific resistance of the sludge. The respective SRFs of CPAM1 and CPAM2 were 6.43×10^12^ and 5.88×10^12^ m•kg^−1^ at 40 mg/L dosage. Addition of CTAB reduced the SRFs of CPAM1 and CPAM2 to 5.93×10^12^ and 5.50×10^12^ m•kg^−1^, respectively. Compared to not using surfactant, The SRFs were reduced by 6.9%–7.8%. SRF is mainly affected by the characteristics of sludge flocs. Reduction in SRF was attributed to the formation of large but strong flocs with narrow size distribution similar to the result of Besra et al. [28] Sludge particles that were too small caused the filtration pores clogged and increase in SRF. The effect of flocculation increased the size of the sludge flocs and formed the rugged flocs construction by adding CPAM; the porous structure of sludge flocs was maintained during filtration and dehydration, thereby reducing the specific resistance of sludge.

The correlation between EPS concentration and SRF was the same as in the previous works on sludge co-conditioning by CTAB and CPAM (Figures 4 and 8) [29]. In addition, the dewaterability of sludge treated with both CPAM and CTAB supplementation was better than that of sludge treated with CTAB or CPAM alone [30], which was attributed to the increase in concentrations of free and unbound EPS, because surfactants could significantly release these substances. EPS and internal bound water were released by adding CTAB, and improved the performance of sludge dewatering.

### Zeta potential

**Figure 9:**
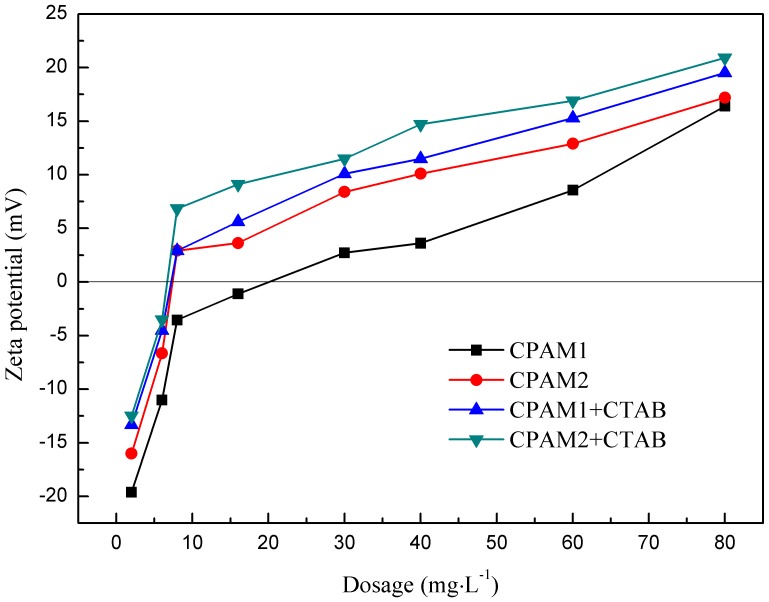
Effect of flocculant dosage on Zeta potential.

The effect of flocculant dosage on Zeta potential is shown in Figure 9; the charge neutralization capacity of the flocculants administered with or without CTAB decreased in the following order: CPAM2+CTAB> CPAM1+CTAB> CPAM2> CPAM1. A low CPAM dosage is necessary to attain zero charge upon addition of CTAB during flocculation; a portion of the negative surface charge on the sludge surface was already neutralized by the cationic CTAB. The Zeta potential of CPAM+CTAB was slightly shifted to a value lower than that of CPAM.

With the increase in flocculant dosage, the Zeta potential significantly increased to an isoelectric point where the potential was close to zero (0 mV). The dosage of the isoelectric point was less than 20 mg/L, while the optimal dosage for sludge dewatering was 40 mg/L. Pefferkorn et al. [31] reported that if charge neutralization was the only flocculation path, the optimal efficiency was achieved when the Zeta potential was close to zero. The potentials treated by CPAM+CTAB at optimal dosages were much higher than the isoelectric point, indicating that charge neutralization insignificantly contributed to sludge dewatering. In contrast to CPAM alone, the Zeta potential significantly increased when CPAM was with CTAB. The addition of CTAB increased the charge neutralization capacity of CPAM, because the former was cationic and neutralized the negative charge on surfaces of sludge flocs. The result implied that the sludge was flocculated by absorption bridging, charge neutralization, and the mechanism of the electrostatic path [32]–[33].

### Floc size

**Figure 10:**
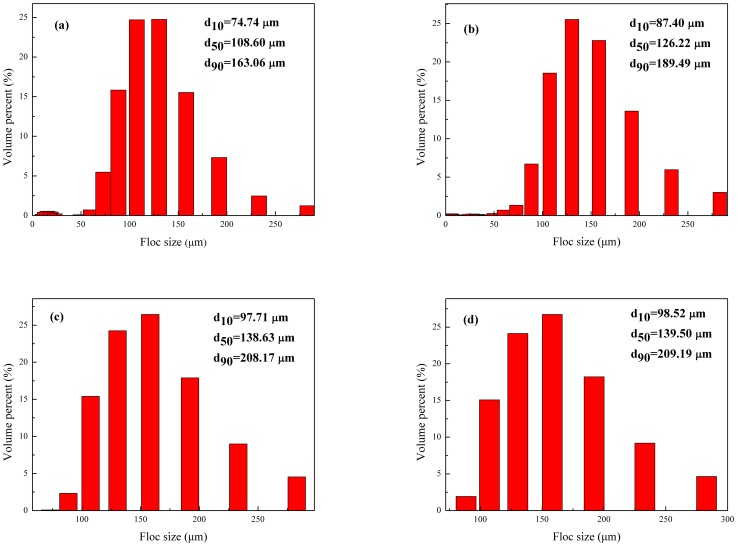
Floc size distribution after flocculation for (a) CPAM1, (b) CPAM2, (c) CPAM1+CTAB, and (d) CPAM2+CTAB.

Figure 10 shows the floc size distributions at the end of flocculation, which were expressed as respective particle sizes corresponding to 10%, 50%, and 90% of the size histogram; 10% of the flocs had sizes in the range [0 µm–d_10_], 50% of the flocs had sizes in the range [0 µm–d_50_], and 90% of the flocs had sizes in the range [0 µm–d_90_]. Larger flocs provide were easier for dewatering than smaller flocs; smaller flocs clog the cake during filtration, thereby reducing dewaterability [17]. The respective values of d_50_ for CPAM1, CPAM2, CPAM1+CTAB, and CPAM2+CTAB with the same dosage at pH 7were 108.6, 126.22, 138.63, and 139.50. Comparison of CPAM1 with CPAM2 implied that a high cationic degree yielded a large particle diameter. Comparison between CPAM1 (CPAM2) and CPAM1+CTAB (CPAM2+CTAB) suggested that the addition of CTAB resulted in large floc sizes at the same conditions with a strong capability for charge neutralization. Addition of CTAB increased the EPS concentration in the supernate (Figures 4 and 7). When comparing Figures 4 and 9, high EPS concentrations in the supernate increased the sizes of sludge floc sizes; EPS significantly affected the size and stability of latter sludge flocs. The dewaterabilities of sludge by CPAM and CTAB showed that the cationic surfactants were adsorbed on the sludge surface by electrostatic and Van der Waals forces; the changes in the characteristics of the sludge flocs were also observed, especially in EPS distribution and floc size [34].

### Effect of addition of CTAB on settling behavior

**Figure 11:**
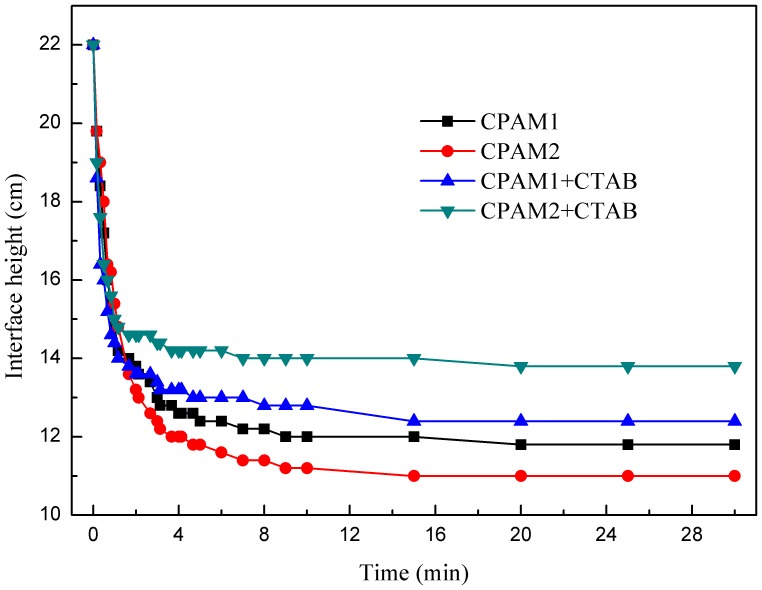
Effect of addition of CTAB on settling behavior.

Figure 11 shows the effect of addition of CTAB on settling behaviors. The settling rate was the average settling rate calculated in the first 2.5 min of settling time. The settling rates for the sludge conditioned with CPAM1, CPAM2, CPAM1+CTAB, and CPAM2+CTAB were 2.79, 3.04, 3.21, and 3.32 cm•min^−1^, respectively. The comparison of CPAM1 and CPAM2 indicated that the flocculants with high cationic degrees yielded rapid settling rates. Compared to not using surfactant, the settling rate was increased by 9.2%–15.1%. The comparison made between CPAM1 (CPAM2) and CPAM1+CTAB (CPAM1+CTAB) revealed that the addition of CTAB hastened the settling rate at the same flocculant dosage; however, the ultimate sediment height flocculated with CTAB was larger than that flocculated without CTAB, reflecting the sludge flocculated without CTAB has better sedimentation properties.

Floc size and EPS mainly affected sludge settling; large floc size of the settlement at the initial stage yielded a rapid settling. The sludge combined with a high EPS concentration worsened the properties of sludge settling. EPS in the supernatant enhanced the flocculation and increased the floc size (Figure 10). The long-chain structure of the cationic surfactant destabilized the colloidal particles by neutralizing the negative charge on the surfaces of sludge flocs [35]. The ultimate height of the sludge-water interface reflected the overall settlement performance. In fact, the relationship between the settling rate of sludge in the first 2.5 min and the ultimate height of sludge-water interface was not very significant. A possible explanation for this opposite phenomenon was that the flocs produced by charge neutralization were always denser and smaller than that generated by the adsorption-bridging effect [36]. According to the discussion about ζ-potential and floc size, the results further demonstrated that the main mechanism flocculated by CPAM1, CPAM2, CPAM1+CTAB, and CPAM2+CTAB was adsorption-bridging effect. However, the charge neutralization ability was strengthened by addition of the CTAB, resulting in generated more denser flocs by CTAB+CPAM in initial stage with rapid settling rate. The settling rate flocculated by CPAM only was lower than that obtained by CPAM+CTAB in the first 2.5 min of settling time. With the increase of the settling time, the adsorption-bridging effect was stronger than the charge neutralization, leading to large and loose flocs. Meanwhile Wang et al. concluded that the settleability of the sludge would be greatly weakened when more EPS (Figure 4) were released in sludge matrix [30]. As a consequence, the ultimate height of the sludge-water interface by CTAB+CPAM was higher than the height by CPAM flocculation.

## Conclusions

The effects of the surfactants on improving the sludge dewaterability by the cationic flocculants were investigated. The experimental results indicated that the addition of flocculants and surfactants in sludge flocculation could improve the dewatering performance in terms of DS content, supernatant turbidity, SRF, floc size, and EPS concentration in the supernatant. DS content dewatered by CPAM without CTAB ranged from 27.3%–28.3%; however, the DS content was increased to 29.5%–34.3% by adding CTAB at pH 3. Sludge sedimentation rate and floc size were significantly accelerated and increased through surfactant application. The improvement of the performance of the sludge dewatering resulting from reduction of EPS and the increase of the floc size in sludge flocculation process. The settling rate flocculated by the CPAM only was lower than that obtained by CPAM+CTAB. However, an opposite phenomenon was observed, the ultimate height of sludge-water interface flocculated by CPAM only was higher than that obtained by CPAM+CTAB. Meanwhile, the sludge flocs were compact, and a large amount of water could be removed during filtration and dewatering. Incorporation of the surfactants had a positive effect on the sludge dewatering.
